# Interaction between NANOS2 and the CCR4-NOT Deadenylation Complex Is Essential for Male Germ Cell Development in Mouse

**DOI:** 10.1371/journal.pone.0033558

**Published:** 2012-03-20

**Authors:** Atsushi Suzuki, Rie Saba, Kei Miyoshi, Yoshinori Morita, Yumiko Saga

**Affiliations:** 1 Interdisciplinary Research Center, Yokohama National University, Yokohama, Kanagawa, Japan; 2 Department of Environment and Natural Sciences, Graduate School of Environment and Information Sciences, Yokohama, Kanagawa, Japan; 3 Division of Mammalian Development, National Institute of Genetics, Mishima, Shizuoka, Japan; Baylor College of Medicine, United States of America

## Abstract

Nanos is one of the evolutionarily conserved proteins implicated in germ cell development and we have previously shown that it interacts with the CCR4-NOT deadenylation complex leading to the suppression of specific RNAs. However, the molecular mechanism and physiological significance of this interaction have remained elusive. In our present study, we identify CNOT1, a component of the CCR4-NOT deadenylation complex, as a direct factor mediating the interaction with NANOS2. We find that the first 10 amino acids (AAs) of NANOS2 are required for this binding. We further observe that a NANOS2 mutant lacking these first 10 AAs (NANOS2-ΔN10) fails to rescue defects in the *Nanos2*-null mouse. Our current data thus indicate that the interaction with the CCR4-NOT deadenylation complex is essential for NANOS2 function. In addition, we further demonstrate that NANOS2-ΔN10 can associate with specific mRNAs as well as wild-type NANOS2, suggesting the existence of other NANOS2-associated factor(s) that determine the specificity of RNA-binding independently of the CCR4-NOT deadenylation complex.

## Introduction

The sexual development of mammalian germ cells leading to the generation of eggs and sperm is a critically important biological process. In the mouse, the primordial germ cells (PGCs) are segregated from the somatic cell lineage at an early gastrulation stage [1]. Although the PGCs are potent precursors for both oogonia and spermatogonia, sexual differentiation is induced after the colonization of the embryonic gonads with somatic cells. Retinoic acid (RA) signaling is implicated as the initial trigger for feminization [2], [3]. RA molecules derived from the mesonephros induce the meiotic initiation of germ cells in female embryonic gonads via the induction of the RA responsive gene *Stra8*, which is required for premeiotic replication [4]. On the other hand, at least two somatic factors are required for masculinization of germ cells in male embryonic gonads. CYP26B1, an RA metabolizing enzyme, is expressed in the Sertoli cells and protects germ cells from exposure to RA, resulting in the suppression of meiosis [2], [3]. In addition, somatically derived fibroblast growth factor 9 (FGF9) promotes the expression of male-type genes including *Nanos2* via its receptors on the surfaces of germ cells [5]. *Nanos2* expression commences by E13.5 after the downregulation of *Cyp26b1* and is required for the maintenance of the male germ cell state [6].

Nanos is an evolutionarily conserved RNA-binding protein that is implicated in germ cell development. Three *Nanos* homologues, *Nanos1–3*, exist in the mouse [7], among which *Nanos2* is expressed only in male gonocytes at the fetal stages and plays a key role in the sexual development of germ cells by suppressing meiosis and promoting male-type differentiation [6]. One of the molecular mechanisms regulating these pleiotropic phenomena is dependent on the interaction between NANOS2 and the CCR4-NOT deadenylation complex [8]. The structure of CCR4-NOT deadenylation complex is also highly and evolutionarily conserved among eukaryotes, consisting of at least 10 CNOT proteins (CNOT1–4, 6, 6L, 7–10) in human and mouse [9], [10]. Among the components of this complex, CNOT1 is the largest protein and acts as a scaffold [11], whereas two different types of deadenylases are contained; CNOT6 or CNOT6L belongs to the exonuclease-endonuclease-phosphatase (EEP) family [12], and CNOT7 or CNOT8 belongs to DEDD (Asp-Clue-Asp-Asp) family, [13]. Although the various functions of this complex have been reported, including transcription, mRNA regulation, and protein ubiquitylation/degradation [9], [14], we focus on the mRNA deadenylation activity since we have previously demonstrated that NANOS2-interacting CCR4-NOT complex retains the deadenylation activity against poly(A) RNA *in vitro* [8]. We expect that the interaction between NANOS2 and CCR4-NOT deadenylation complex may lead the suppression of NANOS2-associated transcripts via deadenylation-mediated RNA degradation. However, the molecular basis underlying this protein interaction remains unknown. In addition, it is also unclear whether or not each of the functions of NANOS2 relies on its association with the CCR4-NOT deadenylation complex.

In our current study, we have explored the molecular basis of the interaction between NANOS2 and the CCR4-NOT deadenylation complex *in vitro* and identified CNOT1 as a direct interacting protein. We further examined the biological significance of this interaction by generating a transgenic mouse that expresses a NANOS2 variant lacking the domain required for its interaction with the CCR4-NOT deadenylation complex.

## Results

### NANOS2 associates with the CCR4-NOT deadenylation complex via a direct interaction with CNOT1

In a previous study, we showed that NANOS2 associates with the CCR4-NOT deadenylation complex in male gonocytes, and that this interaction is responsible for the deadenylation activity of NANOS2 [8]. However, the mechanism underlying this association had remained unknown. To address this issue, we first compared the amino acid sequences of the Nanos proteins among several species from fish to human to screen for possible consensus sequences. Conserved sequences at both the N and C-terminus were identified in addition to two highly conserved CCHC-type zinc finger motifs (Figure S1). The N-terminal sequence was found to be common to all of the species compared whilst the C-terminal sequence was specific to mammals. We thus analyzed the function of the N-terminal conserved sequence given that the CCR4-NOT deadenylation complex is evolutionarily conserved in all of the species compared here.

**Figure 1:**
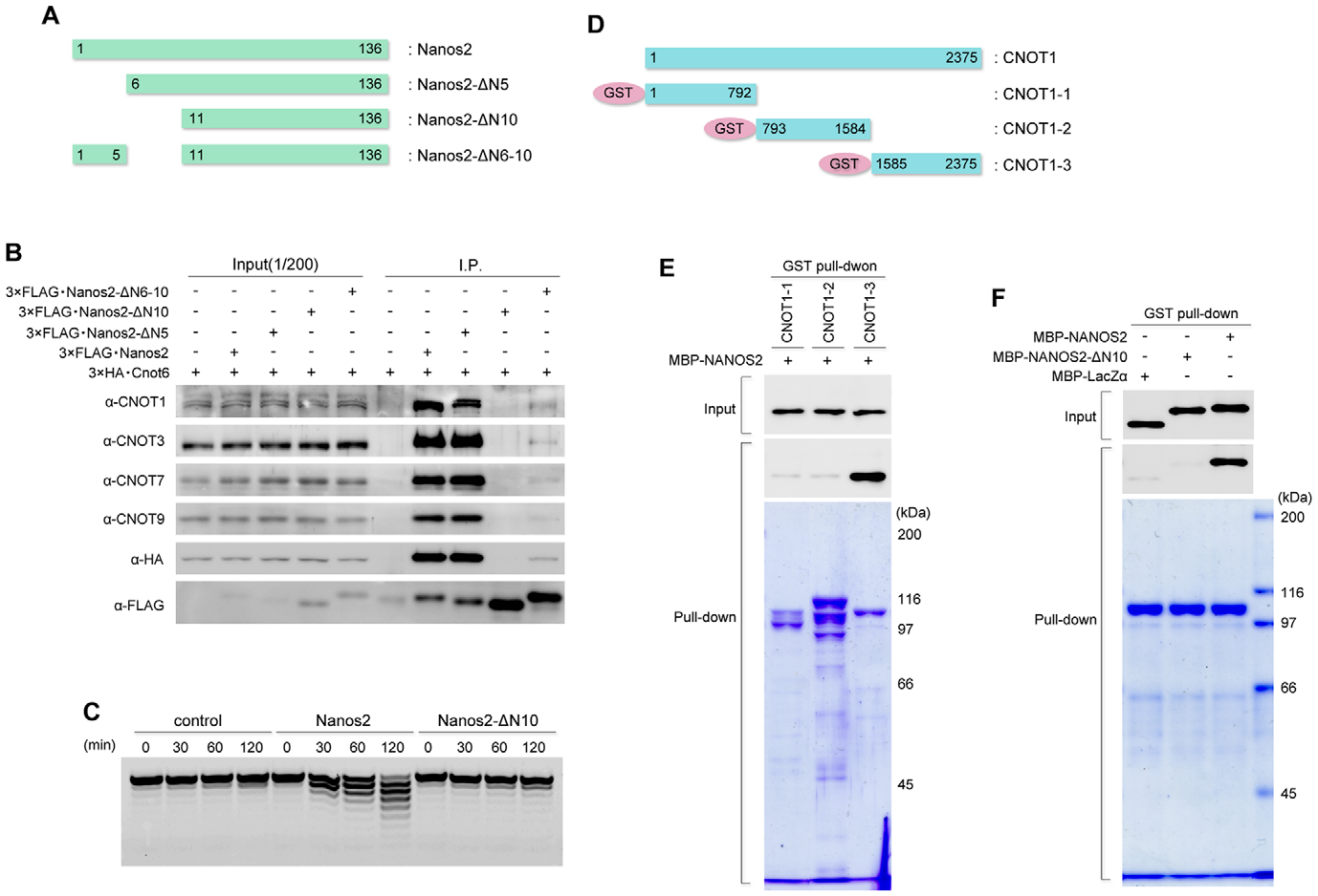
NANOS2 associates with the CCR4-NOT deadenylation complex through a direct interaction with CNOT1. (A) Schematic representation of NANOS2 deletion mutants. (B) Flag-tagged NANOS2 or its deletion mutants were precipitated with anti-FLAG antibodies from HeLa cell extracts co-transfected with 3×HA-Cnot6. Precipitates were analyzed by western blotting with the indicated antibodies. (C) Immunoprecipitated Flag-tagged NANOS2 or NANOS2-ΔN10 were incubated with 5′-fluorescein isothiocyanate-labeled poly(A) RNA substrate for 0, 30, 60 and 120 minutes. Samples were then analyzed on a denaturing sequencing gel. (D) Schematic representation of GST-fused CNOT1 protein. CNOT1 was divided into three parts due to its length: the N-terminal region (CNOT1-1), middle region (CNOT1-2) and C-terminal region (CNOT1-3). (E) *E. coli* extracts expressing GST-fused CNOT1-1, CNOT1-2 or CNOT1-3 were mixed with MBP-NANOS2 and subjected to a GST pull-down assay. (D) *E. coli* extracts expressing CNOT1-3 were mixed with MBP-lacZα, MBP-NANOS2-ΔN10 or MBP-NANOS2 respectively, and subjected to a GST pull-down assay.

We generated several N-terminal deletion mutants of *Nanos2* (Figure 1A) and co-transfected them into HeLa cells with HA-tagged Cnot6, a component of the CCR4-NOT deadenylation complex (since no antibody is currently available). Immunoprecipitation assays revealed that full length NANOS2 efficiently co-precipitated endogenous components of CCR4-NOT deadenylation complex (CNOT1, 3, 7, 9) and also HA-tagged CNOT6 (Figure 1B, lane7), indicating that the interaction between NANOS2 and the CCR4-NOT deadenylation complex can be reproduced in HeLa cells. However, a deletion of the first 10 N-terminal residues of NANOS2 (yielding NANOS2-ΔN10) completely abolished this interaction (Figure 1B, lane 9) whereas there was no such affect if the first 5 amino acids (AAs) were deleted (Figure 1B, lane 8). This indicated the importance of residues 6–10 for this interaction and we generated the corresponding deletion mutant of NANOS2 and found that only small amounts of CNOT protein were precipitated with this product compared with the full length and 1–5 AA-deleted variants (Figure 1B, lane 10). From these data, we concluded that the first 10 AAs of NANOS2 are required for a full interaction with the CCR4-NOT deadenylation complex. We further assayed the deadenylase activity levels of both NANOS2 and NANOS2-ΔN10 using synthetic poly(A) RNA. The resulting data showed that cleavage of poly(A) RNA occurred only with wild-type NANOS2 whereas this activity was not observed in NANOS2-ΔN10 precipitates (Figure. 1C).

We next searched for a direct binding partner of NANOS2 that could mediate the recruitment of the CCR4-NOT deadenylation complex. Given that this interaction can be reproduced even in HeLa cells, we surmised that germ cell specific factors would be unnecessary, which in turn raised the possibility that the direct partner may be one of the components of the CCR4-NOT deadenylation complex. Of note in this regard, *Drosophila* Nanos has been reported to directly bind to CNOT4 in yeast two-hybrid experiments [15]. Hence, we cloned all of the known components of the CCR4-NOT deadenylation complex [16], which include CNOT1–4, 6, 6L, and 7–10, and D1Bwg0212e (a human C2orf29 homologue), into a GST-fusion bacterial expression vector (File S1). CNOT1 was divided into three segments as indicated in Figure 1D because of its long peptide sequence. Following the expression of these components in bacteria, pull-down assays were performed with purified recombinant MBP-NANOS2 (File S1) and revealed that NANOS2 associates only with the C-terminal region of CNOT1 (Figure S2, Figure 1E). We further found that deletion of the 10 N-terminal AAs of MBP-NANOS2 abolishes this interaction (Figure 1F). These data thus revealed that NANOS2 associates with the CCR4-NOT deadenylation complex via a direct interaction with CNOT1.

### The NANOS2-ΔN10 mutant fails to rescue the *Nanos2*-null phenotype

**Figure 2:**
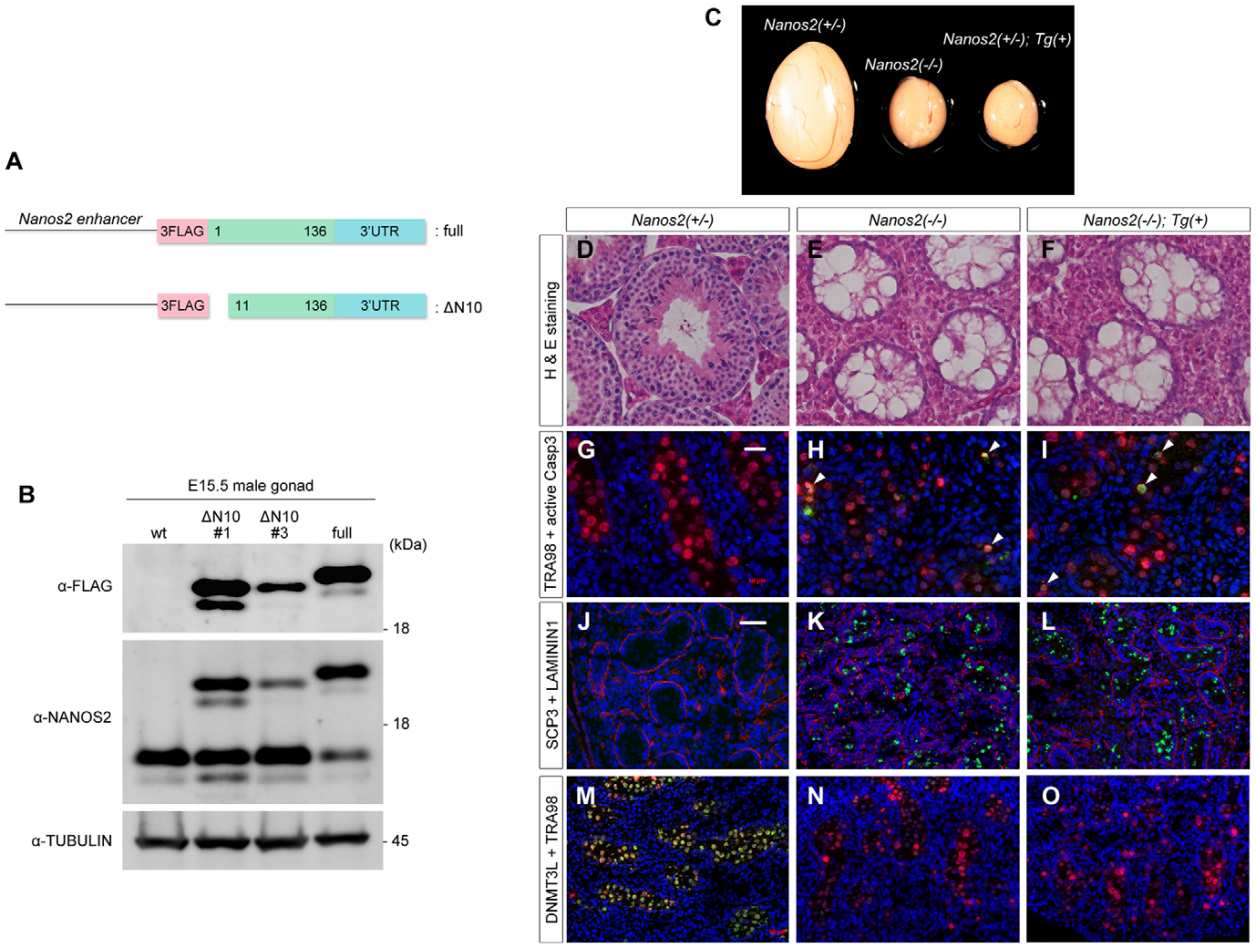
NANOS2-ΔN10 does not rescue the *Nanos2* knockout phenotype. (A) Schematic representation of Flag-tagged *Nanos2* and *Nanos2-ΔN10* transgenes under the direct control of the *Nanos2* enhancer. (B) Western blotting analysis of NANOS2 protein in E15.5 male gonads from Flag-tagged *Nanos2-ΔN10* transgenic mouse lines #1 and #2 with anti-NANOS2 or FLAG antibodies. Wild-type and Flag-tagged full-length *Nanos2* transgenic mice were used as negative and positive controls, respectively. Tubulin was used as a loading control. (C) Comparison of testis size from 6 week-old littermates of *Nanos2* ^+/−^, *Nanos2* ^−/−^ and *Nanos2* ^−/−^ mice expressing the *Nanos2-ΔN10* transgene. (D–F) Sections were prepared from the testes described in (C) and stained with hematoxylin and eosin. (G–I) Sections of testes from *Nanos2* ^+/−^, *Nanos2* ^−/−^ and *Nanos2* ^−/−^ expressing the Flag-tagged *Nanos2-ΔN10* transgene at E16.5 were immunostained with anti-cleaved Caspase 3 (green) and TRA98 (red) antibodies. (J–O) Sections of testes from the same littermates at E15.5 were immunostained with antibodies against SCP3 (green) and LAMININ (red) (J–L), and DNMT3L (green) and TRA98 (red) (M–O). DNA was labeled with DAPI counterstain (blue). Arrowheads in (H) and (I) indicate germ cells undergoing apoptosis. Scale bars, 20 µm in G for G–I; 50 µm in J for J–O.

NANOS2 regulates several aspects of male gonocyte development such as the suppression of meiosis, promotion of male characteristics and suppression of apoptosis [6]. It is not known however whether all of the functions of NANOS2 are mediated by its interaction with the CCR4-NOT deadenylation complex. We thus tried to express NANOS2-ΔN10 in male gonocytes instead of wild-type NANOS2 to further analyze the physiological significance of this association. We generated a transgenic mouse line that expressed Flag-tagged NANOS2-ΔN10 under the direct control of the *Nanos2* enhancer (Figure 2A, ΔN10). We confirmed the expression of this transgene in the embryonic gonads of two lines. Western blotting revealed that the corresponding transgenic mice produced an appreciable quantity of Flag-tagged NANOS2-ΔN10, and that line #1 expressed this truncated protein at levels that were comparable to the full-length Flag-tagged *Nanos2* (Figure 2B, lane ΔN10#1 and full) transgene that can fully rescue the *Nanos2*-null phenotype (Figure 2A, full) [8].

It is noteworthy that the endogenous levels of NANOS2 were found to be unchanged in the presence of Flag-tagged NANOS2-ΔN10, whilst the presence of full-length Flag-tagged NANOS2 reduced endogenous protein expression (Figure 2B, middle panel). This is consistent with a previous report [8] and we thus analyzed the expression of *Nanos2* mRNA in the E14.5 male gonad of each genotype (i.e. wild-type, Tg with full-length *Nanos2*, Tg with *Nanos2-ΔN10*) by RT-PCR (Figure S3, File S1). In the Tg gonads that express full-length *Nanos2*, the mRNA ratio was found to correlate well with that of the protein products (Figure S3B, lane full; Figure 2B, lane full) and the total RNA amount was similar to that of the wild-type gonads (Figure S3C). However, the mRNA ratio did not always reflect the protein amounts in Tg gonads expressing *Nanos2-ΔN10* (Figure S3B, lane ΔN10; Figure 2B, lane ΔN10#1) and the total RNA amount was elevated in comparison with the protein levels (Figure S3D). These results suggest that in the presence of Flag-tagged NANOS2-ΔN10, endogenous *Nanos2* mRNA produces more protein than expected, whereas mRNA from the transgene produces less, indicating that there is an unknown mechanism underlying the regulation of the NANOS2 protein levels independently of transcription. Further analysis was conducted using line #1, which showed higher expression of the *Nanos2-ΔN10* transgene.

We crossed the *Nanos2-ΔN10* transgenic mice with *Nanos2^LacZ/+^* mice to assess the function of NANOS2-ΔN10 in the absence of wild-type NANOS2. Since the *Nanos2-ΔN10* transgene was successfully transmitted via males, we therefore introduced the Flag-tagged *Nanos2-ΔN10* transgene into *Nanos2*-null testes and compared the phenotype with those of *Nanos2*-null mice to further examine the function of NANOS2-ΔN10 *in vivo*. As shown previously, *Nanos2*-null males have significantly smaller testes than their wild-type counterparts, in which no germ cells exist from about 4 weeks [7]. In our current experiments, we similarly observed smaller testes in the transgenic mice with a *Nanos2*-null background (Figure 2C). A subsequent histological study of these transgenic tissues revealed a complete loss of germ cells from the seminiferous tubules (Figure 2D, E, F). We next assessed whether this was due to a failed rescue event during embryogenesis. We performed immunostaining for activated cleaved caspase 3 at E16.5 and found cells undergoing apoptosis, as predicted from the lack of germ cells in the adult testes (Figure 2G, H, I). We further found an upregulated meiotic marker, SCP3 (Figure 2J, K, L) [17], and downregulated male-specific marker, DNMT3L (Figure 2M, N, O) [18]. These phenotypes were almost identical to those observed in *Nanos2*-null mice [6] and we thus concluded that the first 10 residues of NANOS2 are essential for almost all of its functions. These results also suggest that the interaction of NANOS2 with the CCR4-NOT deadenylation complex is essential for its developmental functions, although we cannot exclude the possibility that the association of other factors with the 10 N-terminal AAs of NANOS2 is also critical.

**Figure 3:**
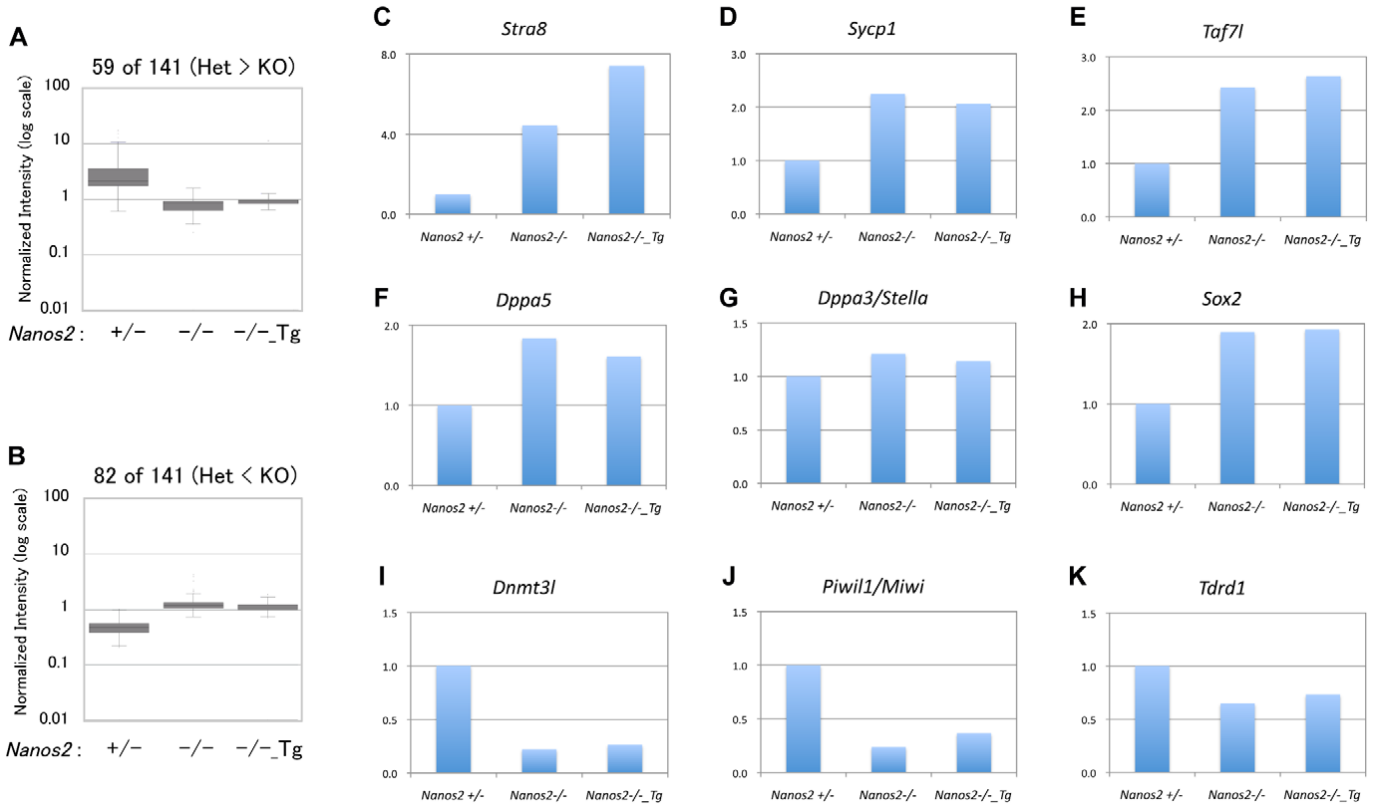
Comparative expression analysis of various genes in *Nanos2* ^−/−^ and *Nanos2* ^−/−^_*Tg^+^* male gonads. (A, B) Box plots showing the expression profiles of 144 genes that are significantly altered in the male gonads of E14.5 *Nanos2* ^−/−^ embryos compared with *Nanos2* ^+/−^ embryos. Note that the averages of the plots for *Nanos2* ^−/−^_*Tg^+^* are very similar to those of *Nanos2* ^−/−^ in terms of both the increased (A) and decreased (B) genes in *Nanos2* ^−/−^. (C–K) Expression levels of genes relevant to the sexual differentiation of germ cells in the male gonads of *Nanos2* ^+/−^, *Nanos2* ^−/−^ and *Nanos2* ^−/−^_*Tg^+^* embryos at E14.5. These data were obtained using the Agilent GeneChip System and analyzed with Genespring GX software.

To further examine the rescue events that cannot be initiated by NANOS2-ΔN10, we compared the gene expression profiles among E14.5 male gonads of *Nanos2^+/−^*, *Nanos2* ^−/−^ and *Nanos2* ^−/−^ mice expressing NANOS2-ΔN10 (*Nanos2* ^−/−^_*Tg^+^*) by microarray. Although a small set of genes showed significant expression changes between *Nanos2* ^−/−^ and *Nanos2* ^−/−^_*Tg^+^* (Table 1), box plot analyses of these genotypes showed that the gene expression profiles were mainly similar, as predicted (Figure 3A–B). Groups of both meiotic genes (*Stra8*, *Sycp1*, *Taf7l*; Figure 3C, D, E) [4], [19], [20] and PGC genes (*Esg1/Dppa5*, *Stella/Dppa3*, *Sox2*; Figure 3F, G, H) [21], [22] were found to up-regulated whilst male-type genes (*Dnmt3l*, *Miwi/Piwil1*, *Tdrd1*; Figure 3I, J, K) [18], [23], [24] were down regulated in E14.5 male gonads as compared with wild-type male gonads, even in the presence of the transgene. These data support our contention that the interaction of NANOS2 with CCR4-NOT deadenylation complex is essential for it to exert its biological roles, and we predict that there are few, if any, CCR4-NOT deadenylation complex-independent NANOS2 functions.

**Table 1:** Results of Microarray analyses.

Subtraction procedures		*Nanos2* ^+/−^	*Nanos2^−^* ^/−^	*Nanos2^−^* ^/−^_Tg^+^	Total
All probe sets		41,326	41,326	41,326	41,326
Present ^a^		25,759	26,036	25,695	27,033*
2 fold change vs *Nanos2* ^+/−^ of 27,033* ^b^		-	804	1,014	-
T-Test, *p*<0.05 vs *Nanos2* ^+/−^ of b ^c^		-	144	219	-
(Ratio of c/*)		-	0.98%	0.81%	-
	2 fold change in c, *<Nanos2* ^+/−^	-	62		
	2 fold change in c, *>Nanos2* ^+/−^	-	82		
2 fold change vs *Nanos2^−^* ^/−^ of 27,033* ^e^		-	-	310	-
T-Test, *p*<0.05 vs *Nanos2^−^* ^/−^ of b ^d^		-	-	37	-
(Ratio of d/*)		-	-	0.14%	-

### NANOS2-ΔN10 interacts with specific RNAs

**Figure 4:**
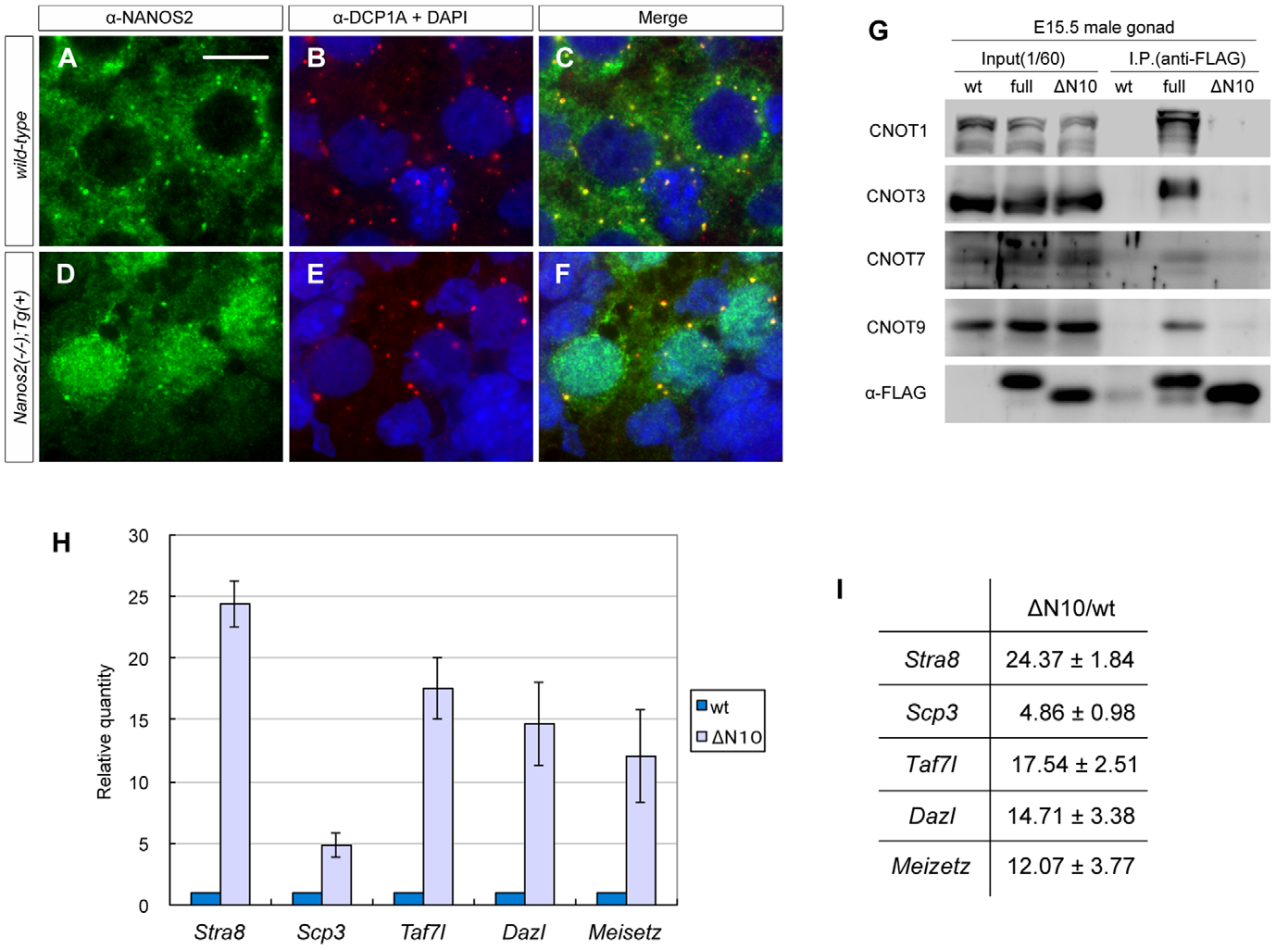
NANOS2-ΔN10 interacts with specific RNAs. (A–F) Sections of testes from *Nanos2* ^+/−^ and *Nanos2* ^−/−^ expressing the Flag-tagged *Nanos2-ΔN10* transgene at E15.5 were immunostained with antibodies against NANOS2 (green) and DCP1A (red). DNA was counterstained with DAPI (blue). Scale bar, 10 µm in A for A–F. (G) Immunoprecipitation-western blotting analysis of proteins from E15.5 male gonadal extracts of wild-type and transgenic embryos expressing 3×FLAG-NANOS2 or 3×FLAG-NANOS2-ΔN10. (H, I) Quantification of mRNA enrichment in 3×FLAG-NANOS2-ΔN10 immunoprecipitates using real-time RT-PCR. The level of *g3pdh* control mRNA was set at 1 and the expression of each mRNA species was calculated relative to this control. The fold enrichment of each transcript in the 3×FLAG-NANOS2-ΔN10 immunoprecipitates compared with wild-type is indicated in the graph (H) and the accompanying table (I). These data were quantified from three QRT-PCR reactions in one set of immunoprecipitations.

To further examine the properties of NANOS2-ΔN10, we next analyzed the cellular localization of this mutant in *Nanos2* knockout mice by immunostaining with the antibody against NANOS2. As previously mentioned, NANOS2 is dispersed throughout the cytoplasm with some localization in P-bodies in male gonocytes (Figure. 4A, B, C) [8]. In contrast to this, however, NANOS2-ΔN10 was mainly found within the nucleus, although was partly still detectable in the cytoplasm and localized at the P-bodies, as seen for wild-type NANOS2 (Figure. 4D, E, F). This indicated that the interaction with the CCR4-NOT deadenylation complex is required for the proper localization of NANOS2. These data raise the question of whether or not its interaction with CCR4-NOT deadenylation complex is also essential for the association of NANOS2 with its target RNAs. To address this issue, we purified FLAG-tagged NANOS2-ΔN10 from E15.5 male gonad extracts to analyze co-precipitated RNA molecules as described previously [8]. Subsequent western blotting analyses revealed that there was no detectable association between NANOS2-ΔN10 and the CCR4-NOT deadenylation complex as the components of which could be efficiently co-precipitated with wild-type NANOS2 (Figure 4G, lane 5) but were undetectable in NANOS2-ΔN10 precipitates (Figure 4G, lane 6). This confirmed that a deletion of the first 10 AAs of NANOS2 abolishes its interaction with CCR4-NOT dedenylation complex *in vivo*.

We also examined the co-precipitated RNAs by real-time RT-PCR, and found that FLAG-tagged NANOS2-ΔN10 efficiently co-precipitated meiotic gene transcripts (*Stra8*, *Sycp3*, *Taf7l*, *Dazl*, *Meisetz*) (Figure 4H–I) that are also associated with wild-type NANOS2 as previously shown [8]. These data indicate that NANOS2 binds specific RNAs independently of its interaction with the CCR4-NOT deadenylation complex.

## Discussion

In our current study, we have identified CNOT1 as a direct NANOS2-associated protein, and shown that the first 10 AAs of NANOS2 is required for this interaction. In addition, we have further shown that the interaction of NANOS2 with the CCR4-NOT deadenylation complex is essential for it to exert its biological roles *in vivo* by using transgenic mouse that expresses a NANOS2 variant lacking these first 10 AAs (NANOS2-ΔN10). As this NANOS2 variant still retains both the CCHC-type zinc finger motif and a C-terminal region highly conserved among mammal (Figure S1B), it is assumed that the *Nanos2-ΔN10* transgenic mouse would have some defects in germ cell development due to presumptive dominant effects. However, we observed normal spermatogenesis and successful transmission of this transgene to next generation, which led us to speculate that endogenous NANOS2 may be sufficient to suppress dominant-negative effects of NANOS2-ΔN10.

On the other hand, NANOS2-ΔN10 did not rescue any major defect observed in *Nanos2*-null mouse, indicating that the NANOS2 function is mediated via interaction with the CCR4-NOT complex. However, it is shown that the complex has various functions other than deadenylation, including transcriptional, post-transcriptional RNA regulation and protein ubiquitylation [9], [14]. For instance, CNOT1 interacts with nuclear receptors [25], and CNOT3 is involved in chromatin remodeling [26], thereby playing a role in transcriptional mechanism. In addition, CNOT4 harbors E3 ligase activity, placing the CCR4-NOT complex in the protein ubiquitylation/degradation pathways [27], [28]. Nevertheless, considered that NANOS2 is cytoplasmic RNA-binding protein localizing in P-bodies and that NANOS2-associated CCR4-NOT complex has deadenylase activity *in vitro* [8], it would be reasonable to assume that a major function of NANOS2-associated CCR4-NOT complex is deadenylation for RNA degradation. However, at this point, we cannot rule out the possibility that some other function(s), such as ubiquitylation or post-transcriptional regulation, is also responsible for a part of NANOS2 function.

We have also shown that a mutant NANOS2 lacking association with CNOT1 still retains an ability to interact with specific mRNAs, indicating that the RNA-binding specificity is determined independently of the interaction with the CCR4-NOT deadenylation complex. However, it is known that the CCHC-type zinc finger motif in NANOS protein binds RNAs non-specifically *in vitro* [29], indicating the other protein(s) is required to confer the specificity. Consistently, our preliminary MAS analyses revealed that several other proteins other than the CCR4-NOT complex are co-precipitated with NANOS2, including several RNA-binding proteins. These factors are currently under investigation.

## Materials and Methods

### Ethics statement

Experiments were carried out with the permission of the animal experimental committee at the Yokohama National University (project number; 1), which is approved March 3, 2009.

### Mice

The *Nanos2*-knockout mouse lines and PCR methods used for the verification of mutant alleles have been previously described [7]. A 3×FLAG-tagged *Nanos2-ΔN10* vector with a 3′-UTR under the control of the *Nanos2* enhancer (9.2 kb upstream sequence) was used for the production of the transgenic mouse line. The primer pairs used for the genotyping of these lines were as follows:

3FLAG-F1; 5′-CTACAAAGACCATGACGGTG-3′, and

N2-3′U-R2; 5′-CCCGAGAAGTCATCACCAG-3′

### Immunoprecipitation and western blotting

The 3×Flag expression vectors for Nanos2 and Nanos2-ΔN10, and 3×HA-Cnot6 were constructed using pcDNA3.1 (Invitrogen). HeLa cells were then transfected with 12 µg of these constructs per 10 cm dish using polyethylenimine [30]. After 48 hours, cellular proteins were extracted with 1 ml of lysis buffer (50 mM Tris-HCl [pH7.4], 150 mM NaCl, 0.5% NP-40, 7.5 mM β-glycerophosphate, 0.1 mM Na_3_VO_4_, 1 mM DTT, 1 mM EDTA, 1 mM PMSF, 1 mM leupeptin, 1 mM aprotinin, 1 mM pepstatin), and spun at 20,000 *g* for 15 min at 4°C. The supernatants were then incubated with 10 µl of anti-FLAG M2 affinity gel (Sigma) on a rotator for 3 h at 4°C. After several washes, precipitates were boiled with 10 µl of 2×Sample buffer, separated by SDS-PAGE, and then subjected to western blotting analysis as described previously. The membranes were incubated with primary antibodies against Flag (1∶8,000; Sigma-F3165), HA (1∶10,000; 12CA5), CNOT1 (1∶500, a gift from H. T. Timmers), CNOT3 (1∶500, a gift from T. Tamura), CNOT7/Caf1 (1∶500, a gift from A. B. Shyu) and CNOT9/Rcd1 (1∶500, a gift from H. Okayama). Positive signals were visualized by incubation with an appropriate secondary antibody conjugated with horseradish peroxidase followed by detection using an ECL Advance™ Western Blotting Analysis System (GE Healthcare). All antibodies were diluted using Can Get Signal Immunoreaction Enhancer Solution (Toyobo).

### *In vitro* deadenylase assay

After immunoprecipitation as mentioned above, precipitates were subjected to a deadenylase assay as previously described [8], [31].

### GST pull-down assay

MBP-LacZα, MBP-NANOS2 or MBP-NANOS2-ΔN10 fusion proteins were expressed in the *E. coli* BL21 (DE3) strain and purified with Amylose Resin (New England Biolabs). All CCR4-NOT deadenylation complex components were cloned from a single stranded E15.5 mouse male gonad cDNA library into pGEX-5X vectors (GE Healthcare), and then expressed in *E. coli*, BL21 Star (DE3) (Invitrogen) cells. Bacterial pellets were sonicated in a binding buffer (25 mM HEPES-KOH [pH 7.4], 150 mM NaCl, 0.1% NP-40, 1 mM DTT, 1 mM EDTA, 1 mM PMSF), and then spun at 15,000 rpm at 4°C. The supernatants were mixed with 1–5 mg of MBP-NANOS2, NANOS2-ΔN10 or LacZα incubated for 2 h at 4°C, and then mixed with 30 µl of glutathione-sepharose 4FF (GE Healthcare) followed by a further incubation for 2 h. After extensive washing with the above binding buffer supplemented with 350 mM NaCl, precipitates were separated by SDS-PAGE and analyzed by western blotting with anti-MBP antibody (1∶2000; New England Biolabs) or by CBB staining.

### Histological methods

For immunostaining, mouse gonads were directly embedded in O.C.T. compound (Sakura) and frozen in liquid nitrogen. After sectioning (8 µm), samples were stained according standard procedures. Details of these methods have been previously described [6].

### Microarray

For one hybridization assay, 200 ng of total RNA was labeled with Cy3 and then hybridized to a Whole Mouse Genome Oligo Microarray (G4122F, Agilent) in accordance with the manufacturer's protocols (Agilent) for the Low RNA Input Linear Amplification Kit, and the One Color Gene Expression Hybridization Kit, respectively. Arrays were analyzed using a Microarray Scanner System (G2565BA, Agilent) and the images were processed with Feature Extraction, version 9.1 (Agilent) to generate signal values and present/absent calls for each probe set. Two independent datasets were obtained for each collation. Processed data were analyzed with Genespring GX software. The following normalization steps were applied to each dataset: measurements were set from less than 5 to equal to 5 for data transformation, per chip normalization was set to the 50^th^ percentile, and per gene normalization was set to median. All data is MIAME compliant and the raw data has been deposited in a MIAME compliant database (GEO, accession number: GSE33138).

### Immunoprecipitation and real-time RT-PCR

For the immunoprecipitation – realtime RT-PCR analysis, 60 male gonads from E15.5 embryos of either wild-type or *Nanos2-ΔN10* transgenic mice were homogenized on ice in 200 µl of Buffer A (25 mM Hepes-KOH [pH7.4], 250 mM sucrose, 75 mM β-glycerophosphate, 1 mM DTT, 0.05% NP-40, 2×Complete Mini (Roche) containing 400 units/ml of RNase inhibitor (Toyobo) and 1/100 volume of phosphatase inhibitor cocktail 1 (Sigma, St Louis, MO), and spun at 10,000 *g* for 10 min at 4°C. NaCl (5 M) was then added to the supernatants to a final concentration of 150 mM. The samples were then mixed with 20 µl of anti-FLAG M2 affinity gel (Sigma) and incubated on a rotator for 3 h at 4°C. After 5 washes with Buffer A containing 150 mM NaCl, co-precipitated RNAs were purified using the RNeasy Mini Kit (Qiagen). After synthesis of first-strand cDNAs with 200 U SuperScript III reverse transcriptase (Invitrogen) and 100 pmol (dT)_20_ primer, real-time RT-PCR analyses were carried out according to manufacture's instruction. The level of *G3pdh* control mRNA was set at 1 and the levels of each mRNA were calculated (each mRNA/*G3pdh* mRNA). Then, the fold enrichment of each mRNA in IP from *tg* extracts compared to IP from *wt* extracts is calculated (ratio of each mRNA level in FLAG IP from *tg* to those from *wt*). Quantifications were from three QRT-PCR in one set of immunoprecipitations [8].

## Supporting Information

File S1 This file includes Materials and Methods for amplification of *cnot* genes and Realtime RT-PCR.
(DOCX)

Figure S1 **Conservation of Nanos proteins.** (A) Amino acid sequence alignment of putative NANOS2 proteins among different vertebrate species. The overall sequence identity values in comparison with the mouse NANOS2 protein are shown at the end of each sequence. Three highly conserved regions are indicated in frame. Red and blue circles indicate conserved CCHC residues in the former and latter zinc finger motifs, respectively. (B) Schematic structure of the NANOS2 protein indicating the conserved zinc finger motif. NR, N-terminal region; CR, C-terminal region.
(TIF)

Figure S2 **GST pull-down assay.** *E. coli* extracts expressing GST-fused CNOT1-1, 1-2, 1-3, 2, 3, 4, 6, 6L, 7, 8, 9, 10, or D1Bwg0212e were mixed with MBP-NANOS2 and subjected to a GST pull-down assay. CNOT proteins that precipitated with Glutathione Sepharose were visualized by CBB staining whereas co-precipitated MBP-NANOS2 was detected by western blotting. Note that only CNOT1-3 precipitates large amount of MBP-NANOS2.
(TIF)

Figure S3 **RT-PCR analyses of** ***Nanos2*** **mRNA.** (A) Schematic representations of endogenous *Nanos2*, *3xFlag-tagged* full-length *Nanos2* and *3xFlag-tagged Nanos2-ΔN10* mRNAs. Red arrows indicate the primer pair used to measure the total *Nanos2* mRNA level (C, D), whilst the blue arrows indicate a primer pair designed to discriminate between endogenous and exogenous *Nanos2* mRNA (B). (B) Semi-quantitative RT-PCR analysis of *Nanos2* mRNA in E14.5 male gonads from wild-type, transgenic mice expressing full-length *Nanos2* and *Nanos2-ΔN10*. (C, D) Comparison between the total *Nanos2* mRNA levels in the E14.5 male gonads of wild-type and transgenic mice expressing full-length *Nanos2* (B) or *Nanos2-ΔN10* (C) by realtime RT-PCR analysis.
(TIF)
