# Supplementary material for: Interaction between NANOS2 and the CCR4-NOT Deadenylation Complex Is Essential for Male Germ Cell Development in Mouse
Source: PLoS One. 2012 Mar 20;7(3):e33558. doi: 10.1371/journal.pone.0033558 (PMC3308992; doi:10.1371/journal.pone.0033558)
Supplement: File S1 — This file includes Materials and Methods for amplification of cnot genes and Realtime RT-PCR. (DOCX) [file pone.0033558.s001.docx]

**Supporting Information Materials and Methods**

**Generation of recombinant NANOS2 and CNOT proteins**

DNA fragment encoding the full-length *Nanos2* was cloned into the pMAL-c2 vector (New England Biolabs). MBP-NANOS2 was then expressed in *E coli*. BL21 (DE3), purified with Amylose Resin (New England Biolabs). DNA fragments encoding the each *Cnot* were amplified by PCR from an E15.5 male gonad cDNA, and cloned into pGEX-5X vectors (GE Healthcare). All the GST-CNOT proteins were then expressed in *E coli*. BL21 Star (DE3) (Invitrogen), pulled-down with Glutathione Sepharose (GE Healthcare). The following PCR primer pairs were used for amplification of each *Cnot* cDNAs;

*Cnot1-1*

Cnot1-1(BamHI)-Fw : atgGGATCCatgaatcttgactcgctc

Cnot1-1(Sal)-Rv : atgGTCGACCTAggccccagttcctatgc

*Cnot1-2*

Cnot1-2(EcoRI)-Fw : atgGAATTCcttggacttcctgcagtg

Cnot1-2(Sal)-Rv : atgGTCGACCTAtgtaggtaagaagccag

*Cnot1-3*

Cnot1-3(Sal)-Fw : atgGTCGACaatgacttaagtcagcct

Cnot1-3(Sal)-Rv : atgGTCGACctaactggcaccggtccc

*Cnot2*

Cnot2(Sal)-Fw : atgAAGCTTatggtgaggactgatgga

Cnot2(Xho)-Rv : atgCTCGAGccaagaaaagggaagtcttt

*Cnot3*

Cnot3(Hind)-Fw : atgAAGCTTatggcggacaagcgcaaa

Cnot3(Xba)-Rv : atgTCTAGAtgtcactggaggtcccggt

*Cnot4*

Cnot4(Sal)-Fw : atgGTCGACatgtctcgcagtcctgat

Cnot4(Xho)-Rv : atgCTCGAGcaattccctctttgcttagt

*Cnot6*

Cnot6(BamHI)-Fw : atgGGATCCatgcccaaagaaaagtac

Cnot6(Sal)-Rv : atgGTCGACctacctcctgccaggaag

*Cnot6l*

Cnot6l(Sal)-Fw : atgGTCGACatgagactaatagggatg

Cnot6l(Sal)-Rv : atgGTCGACctacctccgattaggcaa

*Cnot7*

Cnot7(BamHI)-Fw atgGGATCCatgccagcagcaaccgta

Cnot7(Sal)-Rv : atgGTCGACtcatgactgcttgctgg

*Cnot8*

Cnot8(BamHI)-Fw : atgGGATCCatgcctgcggcacttgta

Cnot8(Sal)-Rv : atgGTCGACtcactgctgcatgttgtt

*Cnot9*

Cnot9(Hind)-Fw : atgAAGCTTatgcacagcctggcaacg

Cnot9(Xho)-Rv : atgCTCGAGtgagagggaacgaggatca

*Cnot10*

Cnot10(Hind)-Fw : atgAAGCTTatggctgcagacaagcctg

Cnot10(Xho)-Rv : atgCTCGAGtcacttcctctgcacggt

*D1Bwg0212e*

D1Bwg0212e(EcoRI)-Fw : atgGAATTCatgcccggcggcggggcgag

D1Bwg0212e(SalI)-Rv : atgGTCGACttattttgatattttggtct

**Real-time RT-PCR**

Total RNA isolates were prepared from the E14.5 male gonads of wild-type, full-length and *Nanos2-ΔN10* transgenic mice using an RNeasy Mini Kit (Qiagen). Aliquots of 0.5 µg of total RNA were then used in a cDNA synthesis reaction with SuperScript III reverse transcriptase (Invitrogen). For real-time RT-PCR, amplification reactions were carried out in 96-well microtiter plate wells in a 25 μl reaction final volume with SYBR premix Ex Taq (Takara), optimized concentrations of specific primers, and a 1:20 dilution of each cDNA as the template. Reactions were carried out using a Dice Real Time PCR Detection System (Takara) and the cycling conditions involved an initial step of 10 s at 94°C, followed by 40 cycles of 15 s at 94°C, 15 s at 60°C, 30 s at 72°C. Each assay was run in triplicate and negative controls (no template or template produced without the RT enzyme) were always included. The data were processed using Thermal Cycler Dice RealTime System Software (Takara) and Microsoft Excel. A normalization factor was calculated using the reference gene *G3PDH*.

The following PCR primer pairs were used for the amplification of total *Nanos2* mRNA: Nanos2-Fw for RT-PCR: attcagagccggaagcaaag**,** Nanos2-Rv for RT-PCR: gactgctgttgagtggacaa. For semi-quantitative RT-PCR, PCR reactions were carried out in 8-well microtiter tubes in a 20 μl reaction volume with Takara-Taq (Takara) with optimized concentrations of specific primers and a 1:20 dilution of each cDNA as template. Products were separated in 11% polyacrylamide gels stained with e[thidium bromide](http://lsd.pharm.kyoto-u.ac.jp/weblsd/c/begin/ethidium%20bromide) and were visualized using a Bio Doc-It^TM^ system (UVP).
