# Supplementary figures and images for: Interaction between NANOS2 and the CCR4-NOT Deadenylation Complex Is Essential for Male Germ Cell Development in Mouse
Source: PLoS One. 2012 Mar 20;7(3):e33558. doi: 10.1371/journal.pone.0033558 (PMC3308992; doi:10.1371/journal.pone.0033558)

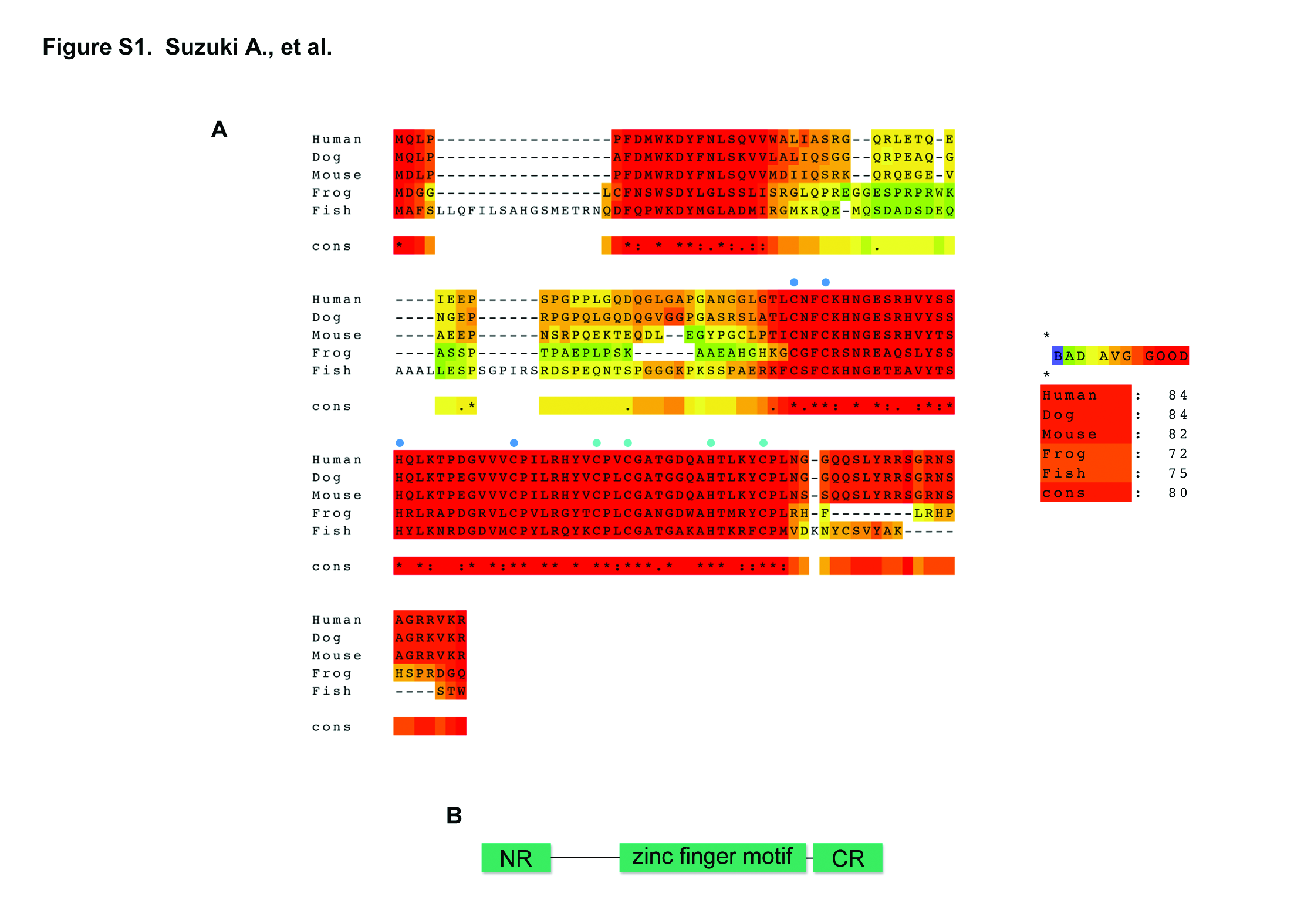

Supplement: Figure S1 — Conservation of Nanos proteins. (A) Amino acid sequence alignment of putative NANOS2 proteins among different vertebrate species. The overall sequence identity values in comparison with the mouse NANOS2 protein are shown at the end of each sequence. Three highly conserved regions are indicated in frame. Red and blue circles indicate conserved CCHC residues in the former and latter zinc finger motifs, respectively. (B) Schematic structure of the NANOS2 protein indicating the conserved zinc finger motif. NR, N-terminal region; CR, C-terminal region. (TIF) [file pone.0033558.s002.tif]

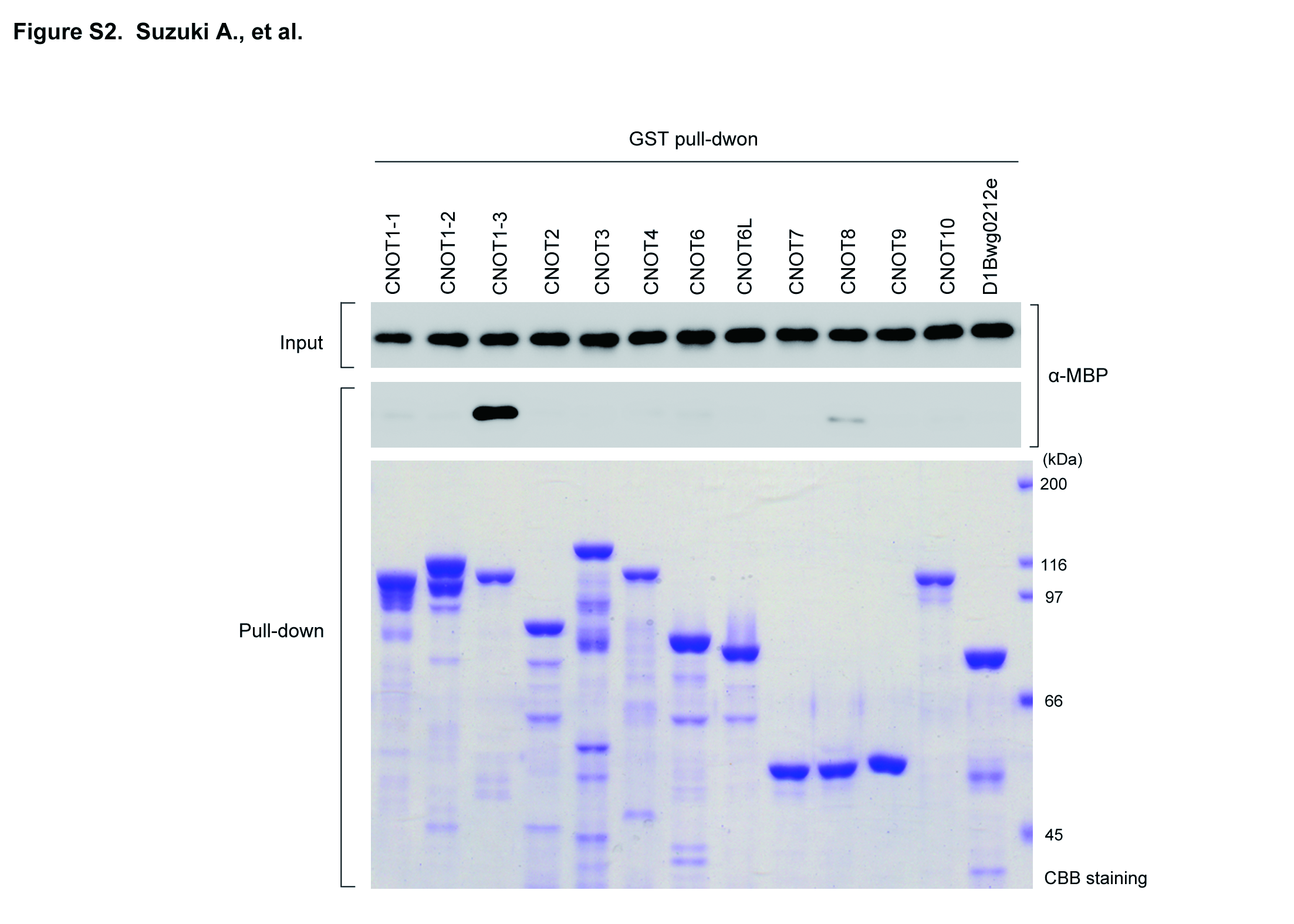

Supplement: Figure S2 — GST pull-down assay. E. coli extracts expressing GST-fused CNOT1-1, 1-2, 1-3, 2, 3, 4, 6, 6L, 7, 8, 9, 10, or D1Bwg0212e were mixed with MBP-NANOS2 and subjected to a GST pull-down assay. CNOT proteins that precipitated with Glutathione Sepharose were visualized by CBB staining whereas co-precipitated MBP-NANOS2 was detected by western blotting. Note that only CNOT1-3 precipitates large amount of MBP-NANOS2. (TIF) [file pone.0033558.s003.tif]

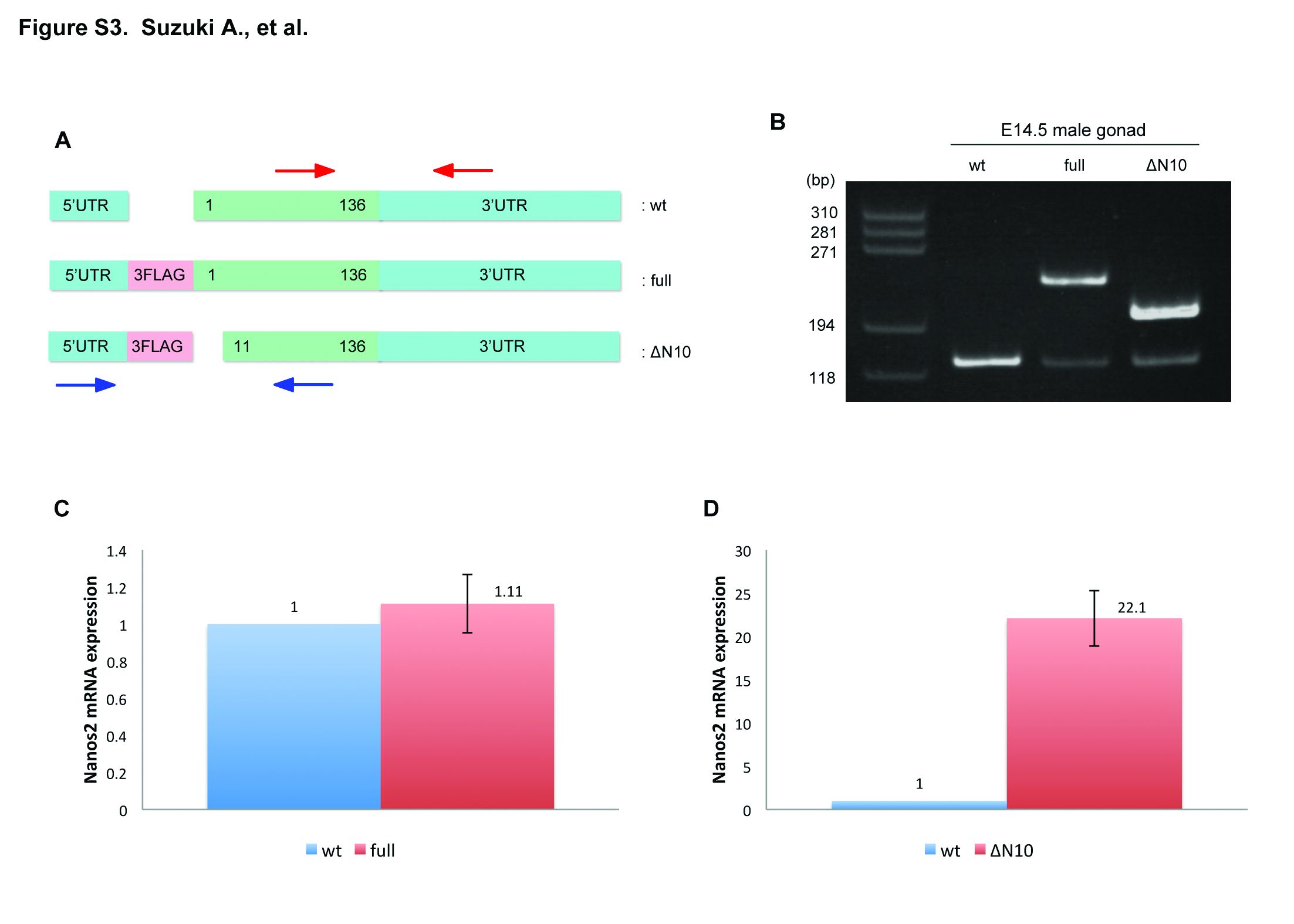

Supplement: Figure S3 — RT-PCR analyses of Nanos2 mRNA. (A) Schematic representations of endogenous Nanos2, 3xFlag-tagged full-length Nanos2 and 3xFlag-tagged Nanos2-ΔN10 mRNAs. Red arrows indicate the primer pair used to measure the total Nanos2 mRNA level (C, D), whilst the blue arrows indicate a primer pair designed to discriminate between endogenous and exogenous Nanos2 mRNA (B). (B) Semi-quantitative RT-PCR analysis of Nanos2 mRNA in E14.5 male gonads from wild-type, transgenic mice expressing full-length Nanos2 and Nanos2-ΔN10. (C, D) Comparison between the total Nanos2 mRNA levels in the E14.5 male gonads of wild-type and transgenic mice expressing full-length Nanos2 (B) or Nanos2-ΔN10 (C) by realtime RT-PCR analysis. (TIF) [file pone.0033558.s004.tif]
